# Tongue image fusion and analysis of thermal and visible images in diabetes mellitus using machine learning techniques

**DOI:** 10.1038/s41598-024-64150-0

**Published:** 2024-06-24

**Authors:** Usharani Thirunavukkarasu, Snekhalatha Umapathy, Vinayakumar Ravi, Tahani Jaser Alahmadi

**Affiliations:** 1 Department of Biomedical Engineering, College of Engineering and Technology, SRM Institute of Science and Technology, Kattankulathur, Tamil Nadu 603203 India; 2 College of Engineering, Architecture and Fine Arts, Batangas University, Batangas City, Philippines; 3 Department of Biomedical Engineering, Saveetha School of Engineering, Saveetha Institute of Medical and Technical Sciences, Chennai, Tamil Nadu 602105 India; 4 Center for Artificial Intelligence, Prince Mohammad Bin Fahd University, Khobar, Saudi Arabia; 5 Department of Information Systems, College of Computer and Information Sciences, Princess Nourah bint Abdulrahman University, P.O. Box 84428, Riyadh, Saudi Arabia

**Keywords:** Type II diabetes mellitus, Thermal tongue image, Visible tongue image, Image fusion, Discrete wavelet transform, Machine learning classifier, Convolutional neural networks, Image processing, Machine learning, Medical imaging

## Abstract

The study aimed to achieve the following objectives: (1) to perform the fusion of thermal and visible tongue images with various fusion rules of discrete wavelet transform (DWT) to classify diabetes and normal subjects; (2) to obtain the statistical features in the required region of interest from the tongue image before and after fusion; (3) to distinguish the healthy and diabetes using fused tongue images based on deep and machine learning algorithms. The study participants comprised of 80 normal subjects and age- and sex-matched 80 diabetes patients. The biochemical tests such as fasting glucose, postprandial, Hba1c are taken for all the participants. The visible and thermal tongue images are acquired using digital single lens reference camera and thermal infrared cameras, respectively. The digital and thermal tongue images are fused based on the wavelet transform method. Then Gray level co-occurrence matrix features are extracted individually from the visible, thermal, and fused tongue images. The machine learning classifiers and deep learning networks such as VGG16 and ResNet50 was used to classify the normal and diabetes mellitus. Image quality metrics are implemented to compare the classifiers’ performance before and after fusion. Support vector machine outperformed the machine learning classifiers, well after fusion with an accuracy of 88.12% compared to before the fusion process (Thermal-84.37%; Visible-63.1%). VGG16 produced the classification accuracy of 94.37% after fusion and attained 90.62% and 85% before fusion of individual thermal and visible tongue images, respectively. Therefore, this study results indicates that fused tongue images might be used as a non-contact elemental tool for pre-screening type II diabetes mellitus.

## Introduction

Diabetes mellitus (DM) is a persistent metabolic disorder characterized by continuous hyperglycemia resulting from impaired insulin secretion in the body^1,2^. As per the International Diabetes Federation (IDF), the global prevalence of diabetes mellitus in 2021 stood at 536.6 million adults (20–79 years), and it is projected to increase to 783.2 million adults by the year 2045^3^. Within Southeast Asia, the prevalence of DM in 2021 was 90 million individuals, and it is anticipated to rise to 152 million by the year 2045, according to estimates. Globally, 352 million people are at risk of type II DM, and 212 million adults with DM have been undiagnosed till now^4^. World Health Organization (WHO) build the global targets for DM in 2022. The target includes by 2030, 80% of population will be diagnosed as DM and 80% of Population with DM will have good control of glycaemia and hypertension^5^.

As outlined by the American Diabetes Association (ADA), the diagnostic criteria for DM include: (1) Fasting blood glucose (FBG) ≥ 126 mg/dl, (2) Postprandial blood glucose (PPBG) ≥ 200 mg/dl, and (3) HbA1c ≥ 6.5%^6–8^. Table 1 represents the aberration table including symptoms and diagnostic criteria for diabetes mellitus. Maintaining controlled glycaemic levels can prevent the microvascular and macrovascular complications related to DM^9–11^. An invasive method is used in the health care centres to diagnose DM and maintain good glycaemic control for the patients. The vein puncture is a laboratory method commonly used to extract the patient’s blood sample for blood glucose analysis. This procedure may result in nerve damage and harm to nearby anatomical structures of the patients^12^. To overcome the minimal invasive procedure, a non-contact, non-invasive method named thermal imaging method is used for various clinical applications for pre-screening the diseases^13–16^.

**Table 1:** Aberration table indicating symptoms and diagnostic criteria.

Symptoms	Diagnostic criteria
Elevated hunger	Biochemical test
Increased thirst	Hba1c ≥ 6.5%
Weight loss	Fasting blood glucose test ≥ 126 mg/dl
Frequent urination	Post prandial ≥ 200 mg/dl
Blurry vision	Random blood glucose test ≥ 200 mg/dl
Extreme fatigue	
Sores that don’t heal	

The ancient Greek method of tongue diagnosis examined the surface of the tongue that depicts some characteristic features which can uncover the functional status of an individual’s inner organs. According to Traditional Chinese Medicine (TCM) and Traditional Korean Medicine (TKM), the tongue is the reflection of the human viscera^17^. In wider acceptance with TCM, East Asian Medicine (EAM) has also reported that the human tongue has been connected to the human body’s internal organs through meridians and the progression of the diseases is reflected on the surface of the tongue^18^. The visual inspection of the human tongue involves four factors such as color, shape, coating, and texture to diagnose the diseases. Alterations in the geometric characteristics of the human tongue body, such as variations in thickness, presence of cracks, and changes in size, can provide insights into an individual's health information. Traditional Chinese Medicine (TCM) relies on unverified theories concerning meridians and qi. Many Western-trained doctors and medical researchers approach TCM practices with skepticism, as there is a lack of substantial evidence supporting their efficacy, and in some cases, indications that a few may cause harm. Additionally, there have been reports of certain Chinese herbs containing elevated levels of heavy metals, including lead, cadmium, and mercury. In recent years, there has been a rise in adverse reactions associated with Chinese herbal medicine and Traditional Chinese Medicine (TCM). Almost 22% of total injuries are attributed to acute liver injury caused by Chinese herbal medicine. Zhang et al. have found the relationship between the various colour shades of the tongue and the internal condition of the human body system^19^. Though the visual inspection of the tongue is non-invasive, comfortable, and convenient to the subjects to diagnose the diseases, it is very difficult to achieve the standardized and reproducible results. To overcome such challenging issues, along with the visual inspection, the thermal patterns and features present on the surface of the tongue could be helpful to diagnose the diseases.

Numerous researchers have implemented machine learning and deep learning techniques in detection of DM using thermographic database^20–22^. Mincu et al. insisted on the developing benchmarks for driving the artificial intelligence (AI) innovation towards the future growth of healthcare perspectives^23^. Advancements in machine learning techniques and AI now empower automated diagnosis of DM at an earlier stage and facilitate self-management of diabetes therapy^24^.

Kumar et al. performed the study on the detection of DM at an earlier stage using digital tongue images^25^. They extracted the color texture and geometric tongue features using a log Gabor filter. They developed a computerized method for classifying the visible tongue images of normal and DM patients based on the extracted texture features and attained an accuracy of 90%. Zhang and Zhang implemented the quantitative analysis on digital tongue images to differentiate between the healthy and diseased images^26^. The images were categorized utilizing a support vector machine (SVM) classifier, yielding an average accuracy of 76.24% in the DM classification. In another study, Zhang et al. demonstrated the experiment to detect diabetes using the digital tongue instrument^27^. They extracted the color and textural features and attained the classification accuracy of 78.77% using SVM classifiers. Meng et al. conducted a study on the digital tongue image to classify healthy and diabetic patients^28^. They used high dispersal neural network for extracting the features. They fed the features into the SVM classifier for the classification and obtained 91.4% accuracy.

Selvarani et al. conducted a study on tongue thermal images to detect diabetes. They analysed the temperature distribution in the tongue region using dyadic wavelet transform and delta segmentation^29^. They pre-processed the image using the stationary wavelet transform to obtain the smoothened image. The authors applied delta color segmentation in the normal and diabetic tongue image. They extracted the minimal statistical features and compared the normal and diabetic tongue images. They limited their study to the segmentation and feature extraction stage itself and have not been involved in the disease classification process. Beck et al. demonstrated a study to investigate the correlation between tongue temperature obtained through infrared thermography (IRT) and tongue color derived from digital images of the tongue, along with pathological observations in cold heat patterns^30^. The researchers measured the average temperature across various tongue regions and scrutinized the regional variations within the seven designated tongue regions. Simultaneously, the Red (R), Green (G), and Blue (B) values were computed from the digital tongue thermogram, transformed into LAB color space, and analysed for the correlation between the digital color image and tongue temperature. Finally, they concluded that tongue temperature is a partial indicator for cold-heat pattern measurements during discharge-related conditions.

Medical image fusion refers to the process of merging multiple images obtained from various imaging modalities into a unified image that incorporates essential informative content while minimizing redundant information. Several researchers have implemented medical image fusion using different imaging modalities based on a wavelet-based approach^31,32^. Ospina et al. developed a software tool for fusing infrared and visible images based on multimodal fusion technique using a hybrid camera system called INVI (Infrared and visual spectrum images) fusion 1.0^33^. The software contains the following features: (1) the intrinsic and extrinsic camera calibration using 2D homography; (2) used in-built automated tool for enhancing thermal and digital images; (3) fusion of infrared and visible facial and machinery images.

The motivation behind studying the fusion of tongue thermogram and digital tongue image lies in the potential benefits it offers in various fields such as healthcare and diagnostics. By combining these two modalities, researchers aim to enhance the accuracy and reliability of tongue-based analysis for detection of diabetes mellitus. Tongue analysis has long been used in traditional medicine systems such as Traditional Chinese Medicine (TCM) and Ayurveda for diagnosing various health conditions. By integrating thermographic data with digital images of the tongue, healthcare professionals can potentially improve the accuracy of diagnosis for diabetes mellitus in a non-invasive manner.

To our knowledge, this study marks the initial endeavour to combine tongue thermography with digital tongue images for diabetes detection. The primary contributions of this proposed study include:

1. The fusion of human thermal and visible tongue images is performed using discrete wavelet transform based on fusion rules.
2. The performance characteristics of the tongue images in the thermal and digital domains before and after fusion are compared using various machine learning classifiers.
3. Image quality metrics are computed for the fused tongue images to select the best fusion rule, which produces minimum mean square error (MSE) and maximum Peak signal to noise ratio (PSNR).
4. Studied the comparative analysis of the classification performance of visible, thermal and fused tongue images between the machine learning classifiers and convolutional neural network (CNN).

## Methodology

### Study subjects

The study protocol received approval from the institutional ethical committee of SRM Medical College Hospital and Research Centre (834/IEC/2015). The written informed consent and detailed questionnaire were obtained from all the enrolled participants (N = 200) to examine their health status before recruiting for the clinical study. After the strict scrutiny of questionnaires, 40 participants with confounding factors such as pregnant or nursing women, cardiovascular problems, renal failures, fever, thyroid disorders, and anemia were excluded from the clinical study. The remaining 160 participants recruited for the clinical study. The blood sample is collected for all the recruited participants in fasting and postprandial (2 h) conditions to measure their glucose profile. We acquired the tongue thermal and visible images from 160 recruited participants. Based on the diabetes diagnostic criteria by ADA^15^, the study subjects (N = 160) are categorized into two groups, namely.

1. Group I: Normal (N = 80), comprised of age and sex-matched subjects with a Male: Female ratio of 1:2, and a mean age ± standard deviation of 41.23 ± 10.82 years.
2. Group II: Type II DM (N = 80), with a Male: Female ratio of 1:2 and a mean age ± standard deviation of 42.95 ± 9.63 years.

**Figure 1:**
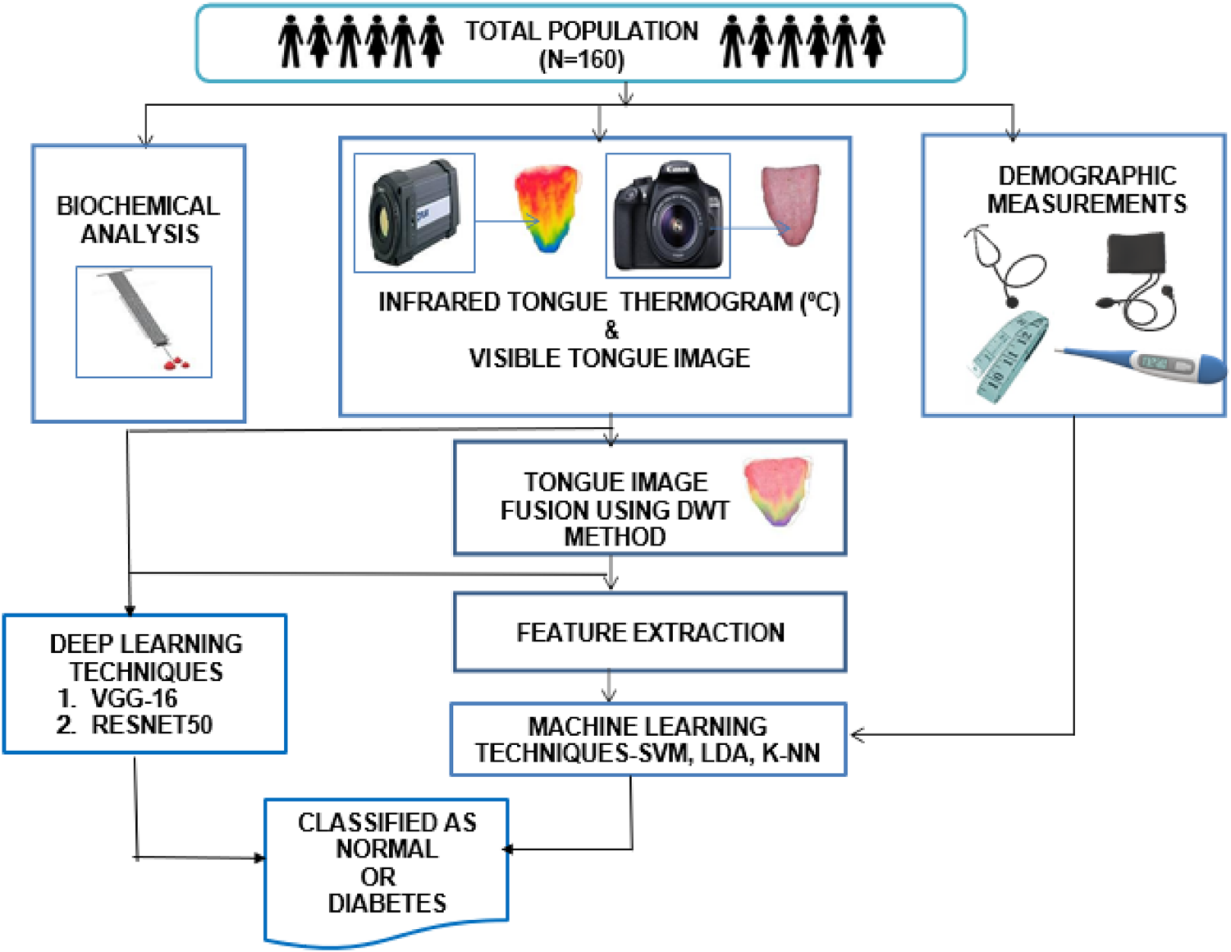
The envisaged study design for pre-screening diabetes mellitus.

The proposed framework for a study focused on pre-screening for Type II diabetes mellitus is as follows (Fig. 1).

### Physiological and biochemical measurements

Anthropometrical variables, including body height (cm), body weight (cm), body mass index (BMI, kg m^−2^), hip and waist circumferences (cm), systolic blood pressure (SBP, mmHg), and diastolic blood pressure (DBP, mmHg), were assessed for all participants. The FBG, PPBG, and HbA1c are the standard biochemical tests performed using participants’ extracted blood samples.

### Thermal and visible tongue images acquisition and analysis

**Figure 2:**
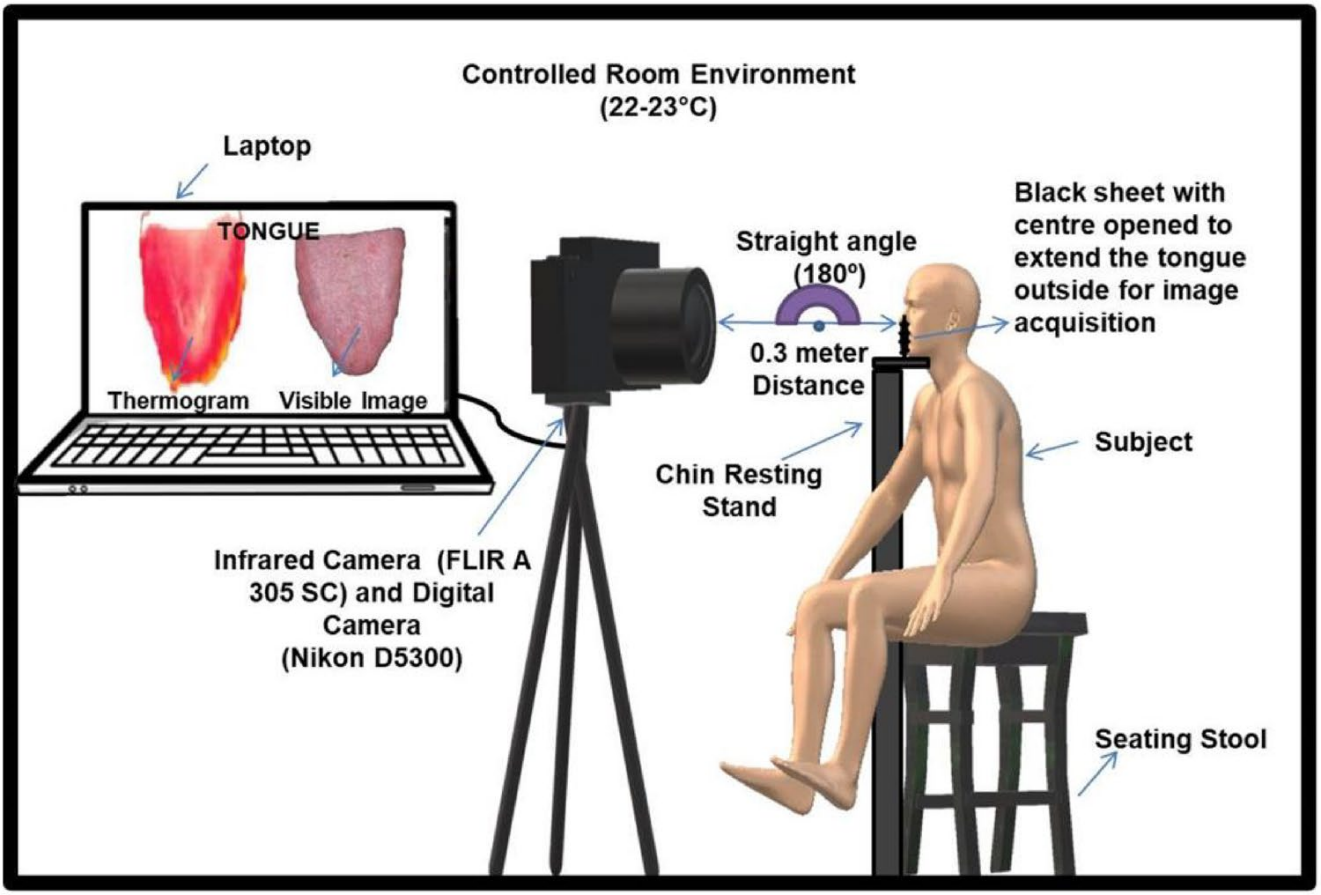
Experimental set up of thermal image acquisition.

From the recruited participants (N = 160), the thermal and visible tongue images are captured using a thermal infrared camera (FLIR A305 SC) and digital single-lens reflex (DSLR) camera (Nikon D5300, 24.2 megapixels), respectively. The FLIR A305SC typically has a thermal resolution of 320 × 240 pixels. It has a thermal sensitivity, typically around 0.05 °C. This means it can detect even small temperature differences accurately. The FLIR A305SC cameras come equipped with an integrated IR lens featuring an 18 mm focal length. The field of view (FOV) is defined as 25° × 18.8°, with an Instantaneous Field of View (IFOV) of 1.36 mrad. These cameras exhibit an accuracy level of ± 2 °C or ± 2% of the reading. The camera was configured with a temperature range of 22–45 °C. We maintained the ambient room temperature between 22 and 23 °C with a relative humidity of 50%. The emissivity of the tongue, when using an infrared thermal camera, is often set to a standard value of around 0.98, corresponding to the emissivity of human skin. The participants are requested to be seated for 15 min to equilibrate themselves with the ambient room temperature. The thermal and visible images are acquired during the fasting condition. The distance between the camera and the subject's tongue is consistently maintained at 0.3 m^34^. Before initiating the imaging process, subjects are instructed to open their mouths widely and extend their tongues downward for 1 min. To prevent artifacts and enhance the background, a black cloth is positioned in front of the patient's mouth. The dimensions of the background were set according to the thermal camera's field of view and the distance between the mouth and the camera. The stabilization time of about 2 min is required between the camera and the patient during the thermal measurements^35^. Skin temperature can be influenced by various factors, including prolonged exposure to specific environmental conditions such as extreme temperatures, humidity, and solar radiation, engaging in extended periods of physical activity^36^. The evaporation of moisture, such as sweat, on the skin's surface can have a significant impact on temperature measurements obtained by a thermal camera. The elevated temperatures be attributed to the reduced evaporation rate, possibly caused by inadequate saliva secretion in tongue of diabetic subject. The thermal tongue images are analysed using FLIR Version 2.0 and MATLAB version R2021a, (Math Works, California, USA) with deep learning package ResNet 50, VGG16. We have chosen the rainbow palette with a constant temperature scale of 28.5–36.9 °C for all the tongue thermograms. According to TCM, the middle portion of the human tongue is connected to the human stomach and Pancreas, as it involves digestion and diabetic conditions^37^. But the link between the middle part of the tongue and the pancreas is significant because it's associated with taste receptors that can help detect sweet flavors, which in turn may influence the release of insulin from the pancreas to regulate blood sugar levels. The upper part of the tongue is associate with kidney. The tip of the tongue is associated with the heart and lungs in TCM and certain alternative health practices. The left and right-side associated regions are related to liver and gall bladder. So, the region of interest (ROI) is positioned at the central part of the tongue (Fig. S1). A square area tool of dimension 50 × 50 pixels is used for analysing the fused tongue images. A square ROI of uniform size of 50 × 50 pixels is fixed semi-automatically on the central region of tongue in visible, thermal and fused tongue images. The ROI is cropped manually and features are extracted using MATLAB programming. The experimental set up illustrates the thermal image acquisition process (Fig. 2).

### Tongue image fusion: DWT method

The fusion of human thermal (FLIR) and visible tongue (DSLR) images are performed using discrete wavelet transform based on fusion rules as follows:

First, the thermal (IR) and visible tongue (DSLR) images need to be pre-processed to ensure they are aligned and have the same dimensions. The thermal image has a real size of 320 × 240 pixels, while the DSLR image has a real size of 390 × 280 pixels. The camera time is not synchronized, with the thermal image captured first and a 2-s delay before acquiring the visible image. Despite potential movement of the tongue between the thermal and visible images, geometric transformations such as translational shifting and rotation (up to 5 degrees) are applied to align the images during the fusion process.

Discrete wavelet transform (DWT) is a mathematical technique employed to decompose an image into various scales and orientations, capturing both high-frequency and low-frequency components. The discrete wavelet transform (DWT) fusion method is often chosen over other fusion techniques due to its ability to efficiently integrate information from multiple sources while preserving important features. DWT allows for multiresolution analysis, which means it can decompose images into different frequency components at varying levels of detail. It enables the representation of both coarse and fine features, capturing a wide range of information. DWT provides a sparse representation of images, which means it concentrates most of the signal energy in a few coefficients. This sparsity property is beneficial for fusion because it facilitates the extraction of relevant information while reducing redundancy. It can localize features in both space and frequency domains. This capability is essential for fusion tasks as it helps preserve the spatial and spectral characteristics of the input data. DWT offers computational efficiency compared to other fusion techniques, especially when dealing with large datasets or real-time processing requirements.

Despite its advantages, the DWT fusion method also presents some limitations and challenges. The DWT has limited directionality because it relies on a predefined set of wavelet basis functions, which may not always capture the directional information present in the input data effectively. The discrete nature of the DWT introduces boundary effects, where discontinuities at the edges of images can lead to artifacts in the fused result. DWT fusion results can be sensitive to scale and shift variations in the input data, particularly when dealing with images acquired under different conditions or sensor configurations. The multiresolution nature of DWT involves a trade-off between resolution and information loss, where higher levels of decomposition offer better frequency resolution but may lead to increased loss of spatial or spectral details.

**Figure 3:**
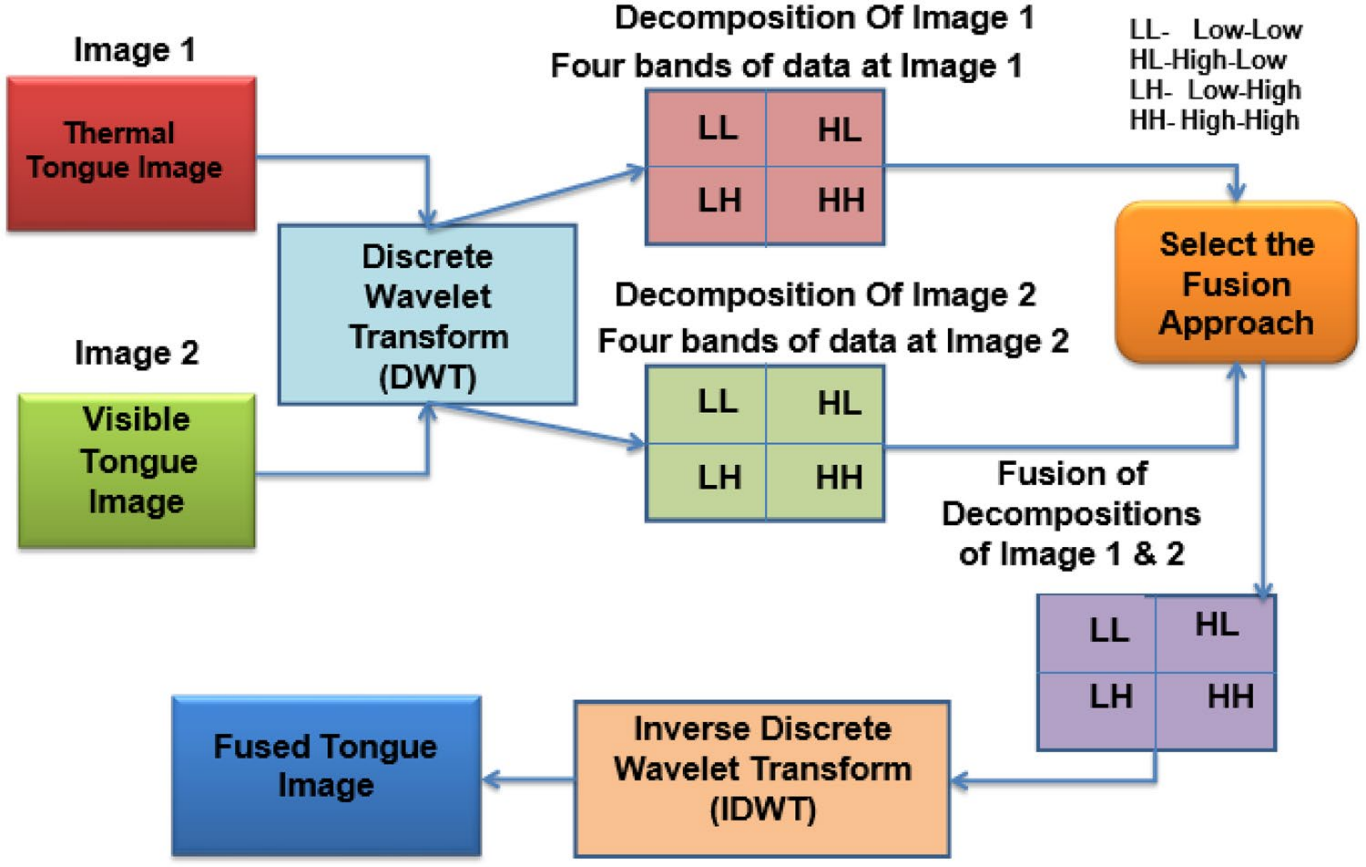
Tongue image fusion using discrete wavelet transform (DWT) method.

Apply DWT separately to the thermal and visible tongue images to obtain their respective wavelet coefficients at different scales and orientations. Fusion rules are used to combine the wavelet coefficients from both images. Example if the rule is Max-Max, select the maximum value of the corresponding coefficients from both images at each scale and orientation. The selection rule is based on choosing the coefficients from one modality based on certain criteria, for example, selecting thermal coefficients for low-frequency components and visible coefficients for high-frequency components. After applying the fusion rule to the wavelet coefficients, perform the inverse DWT to obtain the fused image. This fused image will contain combined information from both the thermal and visible tongue images. The wavelet filter used for tongue image fusion is Daubechies (dB) filter with order two, and the decomposition level is 2. The detailed illustration of tongue image fusion are as follows: (Fig. 3).

The fusion of visible and thermal tongue images is performed based on the fusion rules (Table S1). There are nine different fusion rules for fusing visible and thermal tongue images. For illustration purpose, the proposed study elaborates the mean–max fusion rule. According to the mean–max rule, mean is considered as approximate co-efficient, which contains low frequency information. Max is called detailed co-efficient, containing high frequency level information. The low frequency information generally suppresses the average noise present in the image based on adopting the simple average method. A high frequency component extracts the detailed information related to curves, lines, and contours present in the source image. Hence in the proposed study, the low frequency component from the visible tongue image is fused with the high frequency component from the thermal tongue image. In the end, the reconstructed fused tongue image is acquired through the application of the inverse wavelet transform. The Eq. (1) represents the mean-max fusion rule as given below as follows:

1 $${\text{I}}_{{{\text{fuse}}}} \left( {{\text{x}},{\text{y}}} \right) = {\text{W}}^{ - 1} \left[ {\upphi _{{\left( {{\text{IRT}},{\text{VIS}}} \right)}} \left\{ {{\text{W}}_{{\text{T}}} \left( {{\text{I}}_{{{\text{IRT}}}} \left( {{\text{x}},{\text{y}}} \right)} \right.,{\text{W}}_{{\text{T}}} \left( {{\text{I}}_{{{\text{VIS}}}} \left( {{\text{x}},{\text{y}}} \right)} \right.} \right\}} \right]$$

whereas I_fuse_(x,y)—fused tongue output image, W^−1^—inverse discrete wavelet transform, ϕ—fusion rule mean–max, W_T_—wavelet transform, I_IRT_(x,y)—thermal tongue input image, I_VIS_(x,y)—visible tongue input image.

$$\upphi _{{\left( {{\text{IRT}},{\text{VIS}}} \right)}} = {\text{mean}},\max$$

1a $$\upphi _{{\left( {{\text{IRT}},{\text{VIS}}} \right)}} = {{\left( {{\text{IRT}} + {\text{VIS}}} \right)} \mathord{\left/ {\vphantom {{\left( {{\text{IRT}} + {\text{VIS}}} \right)} 2}} \right. \kern-0pt} 2}\;\,{\text{if IRT}} \ne {\text{VIS}}$$

1b $$\upphi _{{\left( {{\text{IRT}},{\text{VIS}}} \right)}} = {\text{IRT}}\;\,{\text{if IRT}} = {\text{VIS}}$$

The mean-max rule calculates the mean (average) of the two values if IRT and VIS are not equal. It takes the maximum value if IRT and VIS are equal, essentially preserving the maximum value.

According to mean-max fusion rule, mean is derived from approximate coefficient and max is obtained from detailed coefficient

2 $${\text{I}}_{{{\text{fuse}}}} \left( {{\text{x}},{\text{y}}} \right) = {\text{W}}^{ - 1} \left[ {{\text{mean}},\max \left\{ {{\text{W}}_{{\text{T}}} \cdot \left( {{\text{Image}}_{1} \left( {{\text{x}},{\text{y}}} \right)} \right.,{\text{W}}_{{\text{T}}} \cdot \left( {{\text{Image}}_{2} \left( {{\text{x}},{\text{y}}} \right)} \right.} \right\}} \right]$$

### Statistical feature extraction

The thermal, visible, and fused tongue images were converted into grayscale images to extract the statistical features. The Gray level co-occurrence matrix (GLCM) relies on a statistical technique employed to examine texture features, providing insights into the spatial pixel relationships^38–41^. We extracted the statistical parameters such as mean, contrast, standard deviation, correlation, energy, entropy, homogeneity, skewness, variance, and kurtosis from the thermal, visible, and fused tongue images using the GLCM algorithm. The extracted statistical features from the thermal, visible, and fused tongue images were provided (Table S2).

### Machine and deep learning algorithms

#### Machine learning classifiers

The SVM, linear discriminant analysis (LDA), *k*-nearest neighbour (*k*-NN) and Visual Geometry Group Net (VGG16) and ResNet50 were used to perform the classification of diabetes from the fused tongue image. The SVM classifier performs both linear and non-linear (using kernel function) classification. It uses hyperplanes to define the boundaries that exhibit better classification accuracy for minimum datasets. SVM is a binary classifier that maximizes the margin to determine the hyperplane that separates the two classes^42^. LDA is a dimensionality reduction method that separates two or more groups and extends the features in the higher dimensional space to the lower dimension space. It measures various linear features within and between class-scatter matrices^43^. *k*-NN is used for classification and regression problems. It works on the concept that nearer objects are mainly expected to be surrounded by a similar category^44^. In machine learning classifiers, total data used is 160 (80 for diabetic and 80 for Normal). The data split used for training is 70% (112 images), validation is 15% (24 images) and testing is 15% (24 images).

#### Convolution neural network

**Figure 4:**
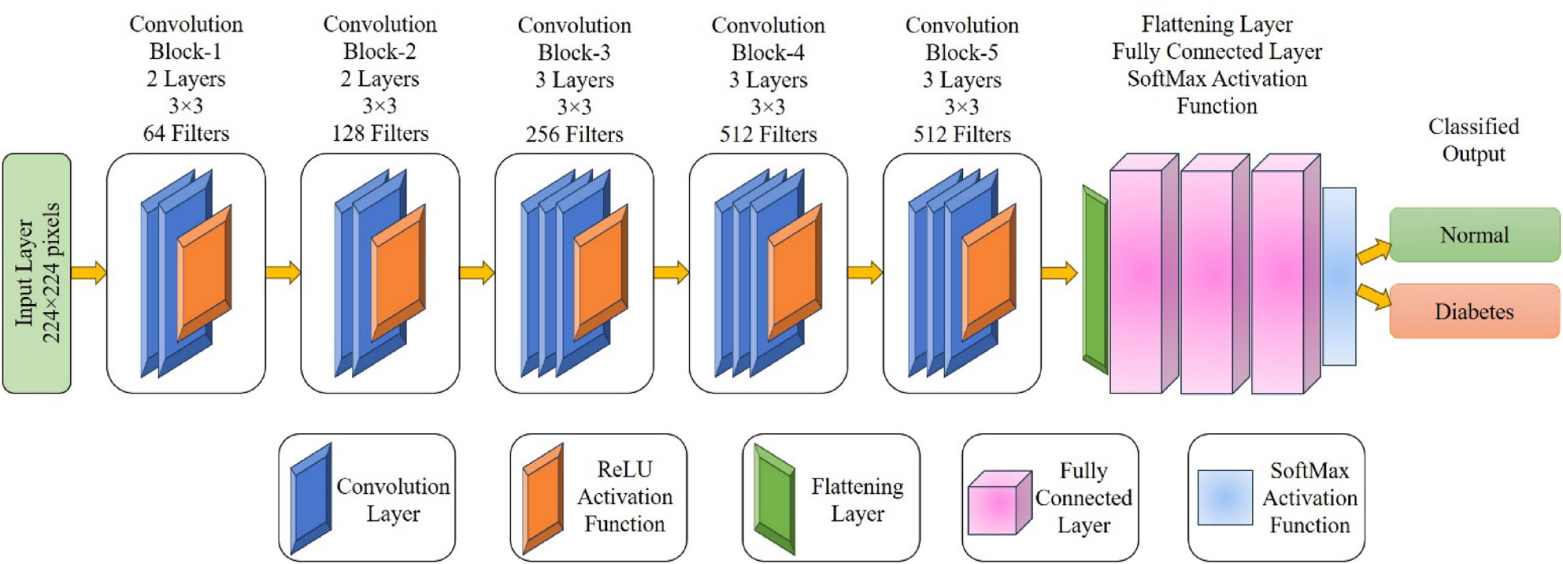
Pre-trained VGG-16 Net for tongue image classification.

Convolutional neural network (CNN) is a type of deep neural network that demonstrates exceptional performance in medical image classification by extracting and learning complex high-level features from the images^45^. The overall architecture of the VGG16 model for Tongue image classification are explained as follows (Fig. 4). VGG16 comprises 13 convolutional layers arranged into five convolutional blocks. Each block consists of multiple 3 × 3 convolutional layers, followed by a max-pooling layer with a pool size of 2 × 2. The convolutional layers are structured to capture various levels of image features, progressively learning more intricate patterns. After the convolutional blocks, VGG16 has three fully connected layers. Each fully connected layer is followed by a rectified linear unit (ReLU) activation function. Before the fully connected layers, there is a flattening layer that converts the 3D feature maps into a 1D vector. The final layer is a SoftMax activation layer, which produces the probability distribution over the different classes. The number of neurons in the output layer aligns with the number of classes in the classification task. The exclusive use of 3 × 3 convolutional filters across the network facilitates a deeper architecture, and the repeated stacking of convolutional layers aids in learning hierarchical features. The stochastic gradient descent (SGD) optimization algorithm is used in VGG16 Net to update the neural network weights with a 0.01 learning rate^46^. Categorical Cross Entropy is employed as the loss function in VGG16.

ResNet50 is a deep CNN architecture that has garnered significant popularity in the field of computer vision, particularly for tasks such as image classification and object detection^47^. The key innovation of ResNet50 lies in the incorporation of residual blocks, aiming to overcome the vanishing gradient problem and facilitate the training of extremely deep neural networks. Residual blocks, the core building blocks of ResNet50, include skip connections, also known as shortcut connections, which bypass one or more convolutional layers. It consists of 48 convolutional layers and 1 fully connected layer, making it a very deep architecture. The input layer accepts input images with a standard size, typically 224 × 224 pixels. The initial layers of ResNet50 comprise standard convolutional layers and pooling layers, designed to extract fundamental features from the input image. The core building blocks of ResNet-50 are residual blocks. Each block contains two or three convolutional layers along with skip connections. Multiple residual blocks are stacked together to form the deeper layers of the network. ResNet-50 includes four stages, each with a different number of residual blocks. After the convolutional layers, a global average pooling layer is used to reduce the spatial dimensions of the feature maps. A fully connected layer processes the output of the global average pooling layer to generate the final classification scores. The SoftMax activation function is then applied to the final layer to transform the raw scores into class probabilities. The output layer provides the final predictions for the input image, indicating the probabilities of different classes. Stochastic gradient descent (SGD) optimizer is used as a hyperparameter in ResNet50 to update the parameters using 0.01 as learning rate. Categorical Cross Entropy is utilized as the loss function in ResNet50.

Google Cloud Platform, commonly known as GCP, is a comprehensive suite of cloud computing services offered by Google. It encompasses a broad array of tools and services for computing, storage, data analytics, machine learning, and more. Google cloud platform is used for training and validation of the CNN model. Dedicated hardware is required for training the neural network weights of the VGG16 net and ResNet50. Hence NVIDIA TESLA V100 based cloud GPU with 16 GB RAM is initiated for the accelerated learning and training process. The CNN models for diabetes detection make use of TensorFlow's image data generator API, specifically version V2.13.0, for efficient execution. TensorFlow, often combined with scikit-learn for machine learning tasks, includes the Keras API for deep learning. OpenCV is commonly used for image processing and computer vision tasks. The fused tongue thermograms are resized to size of 224 × 224 with 3 channels (RGB). The data augmentation techniques such as translational shifting, horizontal flipping, shearing, zooming, and rotational were used to increase the number of inputs thermograms for training deep learning models. As there are five augmentation techniques used, totally 960 images are used after augmentation (160 images (both Normal and diabetes) × 5 = 800 augmented images + 160 original images = 960). Hence the ratio of augmented to original image is 1:6.

#### Data split

The thermogram datasets are divided into three disjoint sets namely training 672 images (70%) used for training, 144 images (15%) used for validation and 144 images (15%) dataset for testing. The same data split is used for both VGG16 model and ResNet50 model. The model is trained for 10 epochs and during each epoch cycle, the CNN will be trained with the train data and gets checked with validation data to get error. Based on this error, the network weights will be varied and retrained for next epoch. Other than weights, other parameters will not be tuned. Early stopping criteria is used to stop the training. The Validation accuracy is used as the performance metric in early stopping with patience = 2. The training process halts when the selected performance measure no longer shows enhancement. Finally, the fully trained model is analysed for the classification performance with the testing dataset.

### Performance evaluation

For the evaluation of image fusion performance, the image quality metrics such as normalized cross-correlation (NCC), mean square error (MSE), peak signal–noise ratio (PSNR), normalized absolute error (NAE), average difference (AD), maximum difference (MD), signal to noise ratio (SNR), structural content (SC) and structural similarity index (SSIM) were used in the proposed study. The definition of image quality metrics^48^ briefed as follows:

Mean Square Error (MSE) quantifies the error of an image by assessing the disparity between the original input image and the processed output image. The lesser value of MSE denotes better performance.

3 $$MSE = \frac{1}{mn}\mathop \sum \limits_{k = 1}^{m} \mathop \sum \limits_{k = 1}^{n} \left( {X_{k,l} - Y_{k,l} } \right)^{2}$$

Peak signal-to-noise ratio (PSNR) is the ratio of the maximum power (peak value) of the information in an image to the power of noise in the image. A higher PSNR value indicates less noise in the image and signifies higher quality in the processed image.

4 $$PSNR = 10\log_{10} \frac{{p^{2} }}{MSE}$$

Normalized cross-correlation measures the degree of similarity between the processed and original images.

5 $$NCC = \mathop \sum \limits_{k = 1}^{m} \mathop \sum \limits_{l = 1}^{n} \frac{{\left( {X_{k,l} *Y_{k,l} } \right)}}{{X_{k,l}^{2} }}$$

Normalized absolute error is similar to MSE, but they have subtle differences in the values.

6 $$NAE = \mathop \sum \limits_{k = 1}^{m} \mathop \sum \limits_{l = 1}^{n} \frac{{\left| {X_{k,l} - Y_{k,l} } \right|}}{{\left( {X_{k,l} } \right)}}$$

Average difference estimates the mean differences between the processed and original images. The value of AD should be as less as possible, and the ideal value is 0.

7 $$AD = 1/mn\mathop \sum \limits_{k = 1}^{m} \mathop \sum \limits_{k = 1}^{n} \left[ {X\left( {k,l} \right) - Y\left( {k,l} \right)} \right]$$

The maximum difference measures the pixel-wise maximum differences between the original and processed output images.

8 $$MD = Max\left( {\left| {X_{k,l} - Y_{k,l} } \right|} \right)$$

Structural content defines the proportion of the sum of the squares of the reference input and processed output image. Let image with the size of m × n matrix

9 $$SC = \frac{{\mathop \sum \nolimits_{k = 1}^{m} \mathop \sum \nolimits_{l = 1}^{n} \left( {X_{k,l} } \right)^{2} }}{{\mathop \sum \nolimits_{k = 1}^{m} \mathop \sum \nolimits_{l = 1}^{n} \left( {Y_{k,l} } \right)^{2} }}$$

whereas m—number of columns, n—number of rows, X—fused or processed image, Y—thermal/visible tongue image, k, l—pixel row and column index.

The structural similarity index (SSIM) is utilized to measure the similarity between two images. It assesses the perceived quality of an image by comparing its structural information to a reference image.

10 $$SSIM = \frac{{\left( {2\mu_{k} \mu_{l} + C_{1} } \right)\left( {2\sigma_{k} l + C_{2} } \right)}}{{\left( {\mu_{k}^{2} + \mu_{l}^{2} + C_{1} } \right)\left( {\sigma_{k}^{2} + \sigma_{l}^{2} + C_{2} } \right)}}$$

where *C*_1_ and *C*_2_ are smoothing constant or regularization parameters

The baseline parameters, anthropometrical variables, tongue temperature, and extracted features from thermal, visible, and fused tongue images are provided as the input to SVM, *k*-NN, and LDA to perform the classification task. The thermal, visible, and fused tongue images were provided directly as input variables to the CNN to perform the classification task. The area under the curve (AUC) was derived from the receiver operating characteristic (ROC) curve of the classifier. The classifiers performance was evaluated by the assessment metrics such as sensitivity, specificity, accuracy, positive predictive value (PPV), and negative predictive value (NPV).

11 $${\text{Sensitivity}} = \frac{TP}{{TP + FN}} \cdot 100$$

12 $${\text{Specificity}} = \frac{TN}{{TN + FP}} \cdot 100$$

13 $${\text{Accuracy}} = \frac{TP + TN}{{TP + FP + TN + FN}} \cdot 100$$

14 $${\text{PPV}} = \frac{TP}{{TP + FP}} \cdot 100$$

15 $${\text{NPV}} = \frac{TN}{{TN + FN}} \cdot 100$$

### Intra- and inter-observer variability

To study the reproducibility and reliability of the imaging process in Tongue thermogram, the intra-observer variability and inter-observer variability was performed using Bland–Altman plot based on temperature measurements from the Tongue thermogram.

In Inter-observer variability, the temperature measurement was made by two different observers using Tongue thermogram. In intra-observer variability, the temperature measurement was made by same observer at different timings to validate the reproducibility.

### Statistical analysis

The data were presented as the mean ± standard deviation (SD). The Shapiro–Wilk test was conducted to assess data normality. To identify significant differences among the groups, the Student's t-test was employed. The data analysis was carried out using SPSS version 21.0 software, Chicago, USA.

### Ethical approval

All procedures performed in studies involving human participants by the ethical standards of the institutional research committee and with the 1964 Helsinki declaration and its later amendments or comparable ethical standards.

### Informed consent

Informed consent was obtained from all participants included in the study.

## Results

The proposed study produced the demographic details such as anthropometrical variables, biochemical parameters, and the measured temperature between the normal and diabetes group (Table 2). The BMI, diastolic blood pressure, biochemical parameters, and measured tongue temperature were highly significant (*p* < 0.01) among the two groups. A mean temperature difference of 1.54 °C was noted between the normal and diabetic patients.

**Table 2:** Demographic variable between the normal and diabetes groups.

Variables	Normal (Group I, N = 80, mean ± S.D = Age = 41.23 ± 10.82 years)	Diabetes mellitus (Group II, N = 80, mean ± S.D = Age = 42.95 ± 9.63 years )	Statistical significance (*p* value)
Anthropometrical			
Waist circumference (cm)	91.71 ± 12.39	93.21 ± 9.01	NS
Hip circumference (cm)	100.11 ± 11.66	103.18 ± 8.22	NS
Body mass index (kg m^−2^)	25.72 ± 4.51	27.78 ± 4.46	*p* < 0.01
Systolic blood pressure (mm Hg)	126.12 ± 15.38	129.50 ± 14.31	NS
Diastolic blood pressure (mm Hg)	80.10 ± 9.21	85.85 ± 10.73	*p* < 0.01
Biochemical			
Fasting blood glucose (mg/dl)	87.30 ± 18.48	148.73 ± 77.94	*p* < 0.01
Post prandial blood glucose (mg/dl)	114.71 ± 15.21	228.45 ± 100.06	*p* < 0.01
Haemoglobin A1c (%)	5.67 ± 0.44	8.67 ± 1.74	*p* < 0.01
Temperature (°C)			
Tongue	34.32 ± 0.42	35.86 ± 0.8	*p* < 0.01

*p* < 0.01—significant, NS—not significant.

**Figure 5:**
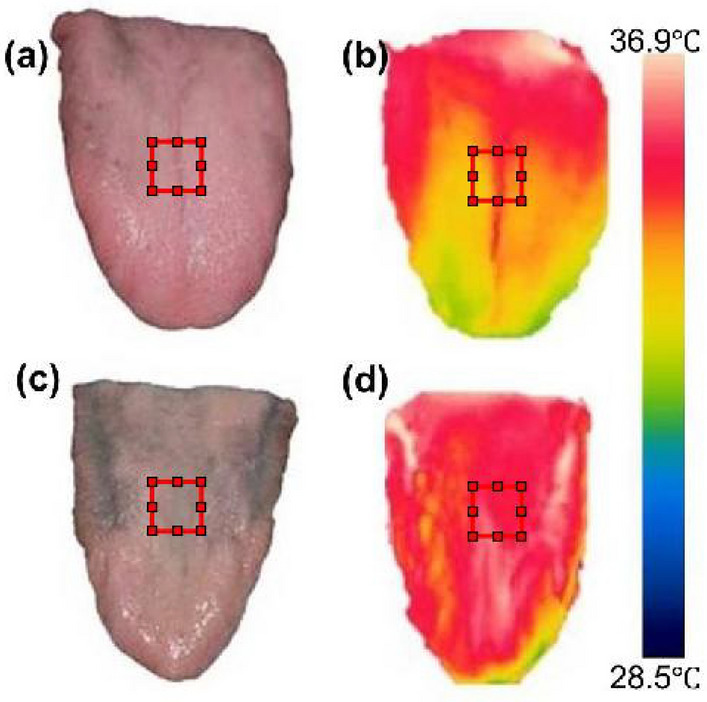
Visible and thermal tongue images acquired from DSLR and FLIR camera. (**a**) and (**b**) normal visible and thermal tongue images, (**c**) and (**d**) diabetic visible and thermal tongue images.

The visible and thermal tongue images obtained from DSLR and FLIR camera (Fig. 5). Figure 5a and c shows the visible image of the normal and diabetic patients. Figure 5b and d indicates the thermal tongue images of the normal and diabetic patients. The increased dark intensity was observed in the visual tongue image for diabetic patients. An elevated temperature was found in the tongue thermogram of diabetic patients due to biological factors such as insufficient secretion of saliva, which leads to xerostomia. We can observe large differences in outer regions apart from ROI in both digital and thermal image. Hence the fusion technique applied to the entire image including the ROI. The result showed good performance only in fused images. When the fused images are given as an input to the machine learning and deep learning classifiers, better accuracy is obtained in VGG16 Net.

**Figure 6:**
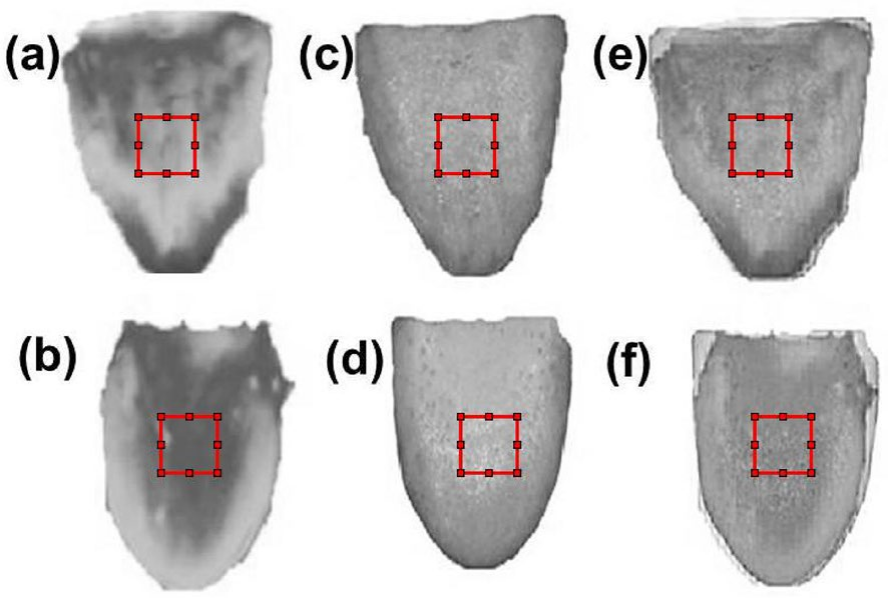
Thermal, visible, and fused (Mean-Max fusion rule) tongue images. (**a**) tongue thermogram of normal subject, (**b**) tongue thermogram of diabetic subject, (**c**) visible tongue image of normal subject, (**d**) visible tongue image of diabetic subject, (**e**) the fused tongue image of normal subject, and (**f**) the fused tongue image of diabetic subject.

The grayscale image of thermal, visible, and fused tongue images of normal and diabetic subjects using the mean-max fusion rule (Fig. 6). Figure 6a and b shows the tongue thermogram of normal subject and diabetic patients respectively. Figure 6c and d indicates the visible tongue image of normal and diabetic patients respectively. Figure 6e and f denotes the fused tongue image of normal subject and diabetic patients respectively.

The statistical features are extracted from the grayscale images of thermal, visible, and fused tongue images for both the normal subject and diabetic patients (Table 3). We found the feature values extracted from the diabetic group to be significantly lower than the normal subjects in tongue thermogram images. The decreased intensity in diabetic thermograms is due to the dryness in the tongue region.

**Table 3:** Extracted Statistical Features using GLCM algorithm from thermal and Visible tongue images before and after Fusion.

Statistical features	Before fusion						After fusion (mean-max rule)		
	Thermal tongue image			Visible tongue image			Fused image		
	Normal (N = 80)	Diabetes (N = 80)	Statistical significance (*p* value)	Normal (N = 80)	Diabetes (N = 80)	Statistical significance (*p* value)	Normal (N = 80)	Diabetes (N = 80)	Statistical significance (*p* value)
Mean	6.17 ± 0.90	6.50 ± 0.41	*p* < 0.01	157.20 ± 25.05	162.01 ± 17.85	NS	206.82 ± 9.73	213.16 ± 11.68	*p* < 0.01
Variance	18.39 ± 8.9	10.38 ± 1.76	*p* < 0.01	167.58 ± 10.43	210.046 ± 16.11	*p* < 0.01	0.008 ± 1.38E	0.0008 ± 1.40E	NS
Energy	0.26 ± 0.17	0.19 ± 0.04	*p* < 0.01	0.52 ± 0.16	0.58 ± 0.18	*p* < 0.01	0.48 ± 0.12	0.64 ± 0.21	*p* < 0.01
Contrast	0.87 ± 0.40	0.75 ± 0.23	*p* < 0.01	0.07 ± 0.03	0.09 ± 0.06	*p* < 0.01	0.13 ± 0.02	0.07 ± 0.04	*p* < 0.01
Correlation	0.78 ± 0.04	0.76 ± 0.08	NS	0.73 ± 0.10	0.80 ± 0.05	*p* < 0.01	0.51 ± 0.19	0.55 ± 0.13	*p* < 0.01
Entropy	45.88 ± 5.12	41.63 ± 10.55	*p* < 0.01	156.11 ± 24.99	160.95 ± 17.32	NS	5.34 ± 0.25	4.84 ± 0.51	*p* < 0.01
Homogeneity	0.82 ± 0.07	0.79 ± 0.02	NS	0.93 ± 0.11	0.95 ± 0.02	NS	0.95 ± 0.01	0.92 ± 0.02	*p* < 0.01
Standard deviation	47.76 ± 18.4	79.91 ± 11.01	*p* < 0.01	12.37 ± 3.65	13.48 ± 5.39	NS	10.66 ± 1.97	8.33 ± 3.48	*p* < 0.01
Kurtosis	0.01 ± 0.006	0.02 ± 0.008	*p* < 0.01	11.51 ± 6.67	10.56 ± 5.77	NS	8.13 ± 7.15E	8.13 ± 7.15E	NS
Skewness	3.77 ± 0.8	2.82 ± 0.35	*p* < 0.01	1.65 ± 1.06	1.38 ± 1.17	NS	− 2.38 ± 2.68E	− 2.38 ± 2.68E	NS

*p* < 0.01 is significant, NS—not significant.

The mean, variance, energy, contrast, energy, and kurtosis parameters exhibit statistically significant differences (*p* < 0.01) between normal and diabetic patients. For visible tongue images, the feature values are elevated in the diabetic group compared to the normal subjects, attributed to the development of a white coating in the upper to the middle portion of the tongue region. The white-coloured layer turns into a yellowish-white layer in the tongue of the diabetic patients as the disease progresses. The fusion of tongue thermogram and visible image results in decreased contrast and entropy. This may be due to reduced intensity level after fusion. But there is no significant difference observed for other parameters such as mean, variance, correlation, energy, and kurtosis.

The thermal and visible tongue images were fused using the nine different coefficient combinations of DWT. The statistical features were extracted from nine coefficient combined fused tongue images based on DWT (Table S2). The mean-max coefficient combination of DWT fusion rules was more significant among the groups and could be used as the best fusion rule of DWT for tongue image fusion (Table S3).

The quality metric analysis was performed before and after the tongue image fusion (Table 3). Before the tongue image fusion, smaller PSNR values and higher MSE and NCC values was observed between the normal and diabetic groups. The higher MSE values indicate the poor image quality and do not provide useful information for further analysis. After the visible tongue image fusion with tongue thermogram, MSE and NCC value was decreased and achieved a higher PSNR value for both the study groups (Table 4).

**Table 4:** Quality Metrics assessment before and after tongue image fusion.

Quality metrics	Before fusion		After fusion	
	Normal (N = 80)	Diabetes (N = 80)	Normal (N = 80)	Diabetes (N = 80)
Mean square error (MSE) ↓	1131.60	1032.27	180.47	233.36
Peak signal noise ratio (PSNR) ↑	17.88	18.17	25.65	24.57
Signal to noise ratio (SNR) ↑	17.30	17.60	25.01	23.95
Normalized absolute error (NAE) ↓	0.055	0.053	0.021	0.026
Normalized cross correlation (NCC) ↓	1.00	1.00	1.00	0.09
Average difference (AD) ↑	− 4.94	− 2.09	− 1.05	− 0.50
Maximum difference (MD) ↓	173.52	142.36	100.61	97.08
Structural content (SC)	0.97	0.98	0.99	0.99
Structural similarity index (SSI) ↑	0.84	0.84	0.92	0.94

The nine fusion rules based on DWT of image quality metrics were compared, and observed that the mean-max fusion rule had obtained lower MSE and higher PSNR values than the other fusion rules (Table S4). The effect of noise was found to be lesser when the PSNR values were higher, indicating the richness of information in the fused tongue image. The mean-max fusion rule of DWT was observed as the supreme fusion rule for thermal and visible tongue image fusion.

**Figure 7:**
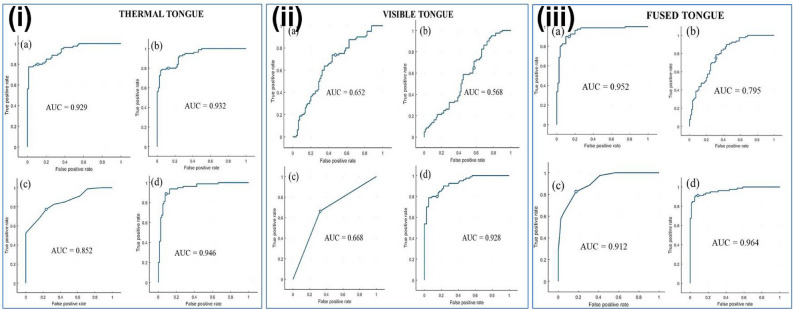
ROC curves of machine and deep learning techniques (1) thermal, (2) visible, and (3) fused tongue images. (**a**) SVM, (**b**) LDA, (**c**) k-NN, and (**d**) CNN-VGG16.

The demographic variables and the extracted statistical features from the visible, thermal tongue, and fused tongue images (using mean-max fusion rule of DWT) of normal and diabetic subjects were given individually to the machine learning algorithms. The deep learning algorithm was fed with fused tongue images generated using the mean-max fusion rule as input for the classification task between normal and diabetic subjects. The classification accuracy of the machine and deep learning algorithms were examined when thermal, visible, and fused tongue images are applied (Table 5). SVM outperformed the machine learning classifiers such as LDA and *k*-NN, well after fusion with a classification accuracy of 88.12% compared to before the fusion process (Thermal-84.37%; visible-63.1%). VGG16 produced a classification accuracy of 94.37% after fusion and attained 90.62% and 85% before the fusion of thermal and visible tongue images, respectively. The ROC curve indicates the performance of classifiers using thermal, visible, and fused tongue images of the total population studied (Fig. 7). After the fusion of visible and thermal tongue images, the area under the curve (AUC) values are better in SVM, k-NN, and VGG16.

**Table 5:** Classification performance of machine and deep learning classifier for diabetes detection.

Tongue images	Machine and deep learning algorithms	ROC–area under curve (AUC)	Sensitivity (true positive rate) %	Specificity (true negative rate) %	Positive predictive value %	Negative predictive value %	Accuracy %
Thermal	SVM	0.929	88.75	80	81.60	87.67	84.37
	LDA	0.932	87.5	80	81.39	86.48	83.75
	k-NN	0.852	76.25	77.5	77.21	76.54	76.87
	VGG16	0.946	90	91.25	91.13	90.12	90.62
	RESNET 50	0.710	74	76	78	76.5	72
Visible	SVM	0.652	52.5	73.75	66.66	60.82	63.1
	LDA	0.568	42.5	65	54.83	53.06	53.75
	k-NN	0.668	67.5	66.25	66.66	67.08	66.87
	VGG16	0.928	82.5	87.5	86.84	83.33	85
	RESNET 50	0.632	63.5	65.25	64	66	65
Fused	SVM	0.952	82.5	93.75	92.95	84.26	88.12
	LDA	0.795	67.5	76.25	73.97	70.11	71.87
	k-NN	0.912	87.5	81.25	82.35	86.66	84.37
	VGG16	0.964	90	98.75	98.63	90.80	94.37
	RESNET 50	0.816	74	75	77	76	78

**Figure 8:**
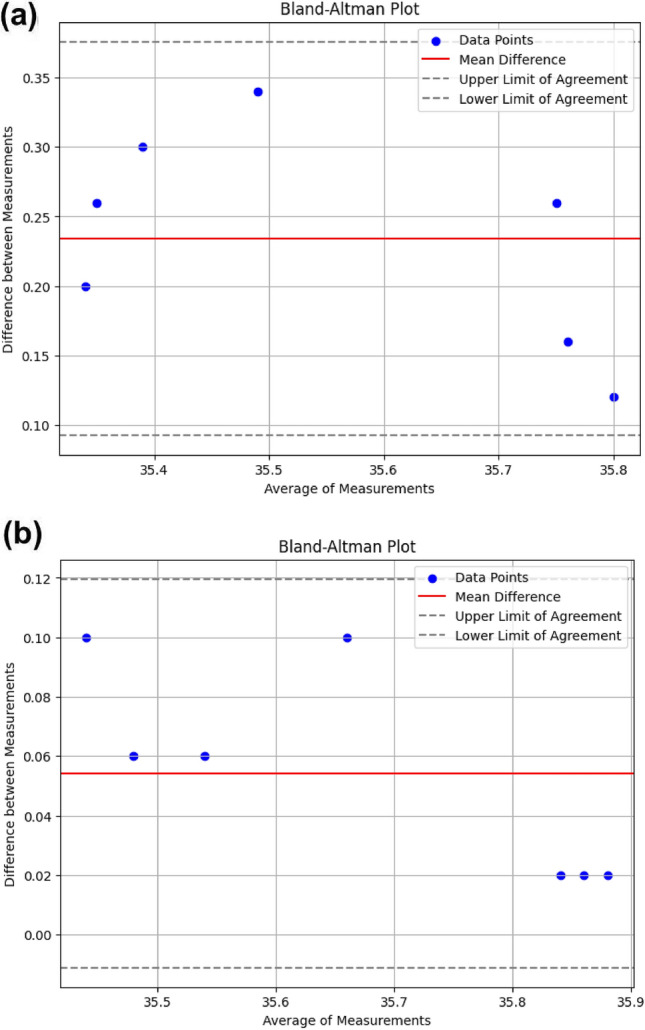
(**a**) Inter-observer variability was performed using Bland–Altman plot based on temperature measurements from the Tongue thermogram. (**b**) Intra-observer variability was performed using Bland–Altman plot based on temperature measurements from the tongue thermogram.

The red line indicates the mean difference between the two measurement methods, which is approximately 0.25 (Fig. 8a). This suggests that, on average, one method consistently measures about 0.25 units higher than the other. The dashed lines represent the limits of agreement, which appear to be around 0.35 (upper limit) and 0.15 (lower limit). These limits indicate the range within which 95% of the differences between the two measurements are expected to lie. The blue dots represent the individual differences between the two methods for each subject. The differences are somewhat spread across the range but mostly fall within the limits of agreement. The mean difference of 0.25 indicates a moderate systematic bias, with one method consistently measuring higher than the other. The limits of agreement range from 0.15 to 0.35. This range shows the extent of variability in the differences. However, the spread of differences suggests that the agreement between the methods has a moderate level of variability.

The mean difference is approximately 0.06 (Fig. 8b). This suggests that on average, one method consistently measures about 0.06 units higher than the other. The dashed lines represent the limits of agreement, which are approximately 0.12 (upper limit) and close to 0.00 (lower limit). These limits indicate the range within which 95% of the differences between the two measurements are expected to lie. The blue dots represent the individual differences between the two methods for each subject. The differences appear to cluster at certain average values. The limits of agreement range from 0.00 to 0.12. This range shows the extent of variability in the differences. In summary, the Bland–Altman plot indicates that while there is a slight systematic difference between the two methods, the overall agreement is quite good, with all differences falling within a narrow and acceptable range.

## Discussion

In this proposed study, the fused thermal and visible tongue images are categorized into the normal subjects and diabetes patients. We used the nine different combinations of DWT coefficients to merge the thermal and visible tongue images. The statistical variables were obtained from the thermal, visible, and fused tongue images using the GLCM algorithm. Among the nine fusion rules, the mean-max and min-mean fusion rules of DWT were statistically significant among the groups. The mean-max fusion rule has obtained lower MSE and higher PSNR values compared to other fusion rules of DWT. Further, the fused tongue images are directly given as the input variables to the VGG16 for the classification task. Demographic parameters extracted from the fused tongue images are given as inputs to the machine learning classifiers.

Cao et al.^49^ have performed the image fusion in diabetic foot images using DWT and Laplace pyramid transform. They applied the mean-max fusion rule with various wavelets such as haar, Daubechies 2, symlets 4, coiflets 2, and biorthogonal 5.5 with the different decomposition levels such as 3, 4, and 5. Image quality metrics were assessed for the fused diabetic foot image employing various wavelets. They achieved superior performance using the mean-max fusion rule with a decomposition level of 5 using haar wavelet transform. Similarly, our study applied the nine different fusion rules of DWT for tongue image fusion to classify diabetes. Among the nine different fusion rules, the mean-max fusion rule has outperformed the other with a minimum decomposition level of 2 using dB2 wavelet transform.

Eid et al.^50^ have performed the fusion process using thermal foot images for the early diagnosis and classification of diabetes mellitus. They have obtained five-hundred-foot thermograms of the total population studied and divided into five different groups according to the grades as follows: Group 1: Grade 0-DM patients without any complications; Group 2: Grade 1-Superficial ulcer formation; Group 3: Grade 2-Deep ulcer formation; Group 4: Grade 3-Amputation patients, and Group 5: Healthy subjects. The histogram and textural features were extracted from the thermal foot images and fused using a concatenating fusion method. They used SVM, *k*-NN, and decision tree classifiers for their classification task. Their experimental result shows that the *k*-NN classifier has achieved the highest classification accuracy rate as 96.8% than other classifiers.

The statistical textural parameters such as contrast, angular moment, entropy, and mean from the standardized visible tongue images of diabetic and healthy subjects were extracted by Zhang et al.^27^. The extracted parameters were given as the input attributes to the developed genetic algorithm (GA) based SVM, *k*-NN, back propagation neural network, and Naïve Bayes to diagnose diabetes mellitus. They obtained the maximum prediction accuracy rate of 79.72% from the developed genetic algorithm (GA)-SVM than the other classifiers for classifying the diabetes patients and normal subjects using the visible tongue images. In the current study, the statistical features extracted from visible tongue images were provided as input to the SVM, k-NN, and LDA classifiers. The visible tongue images are directly delivered as input attributes to the CNN. We found the overall accuracy to be higher in the CNN-VGG16 (85%) than ResNet50 (65%), SVM (63.1%), *k*-NN (66.87%), and LDA classifiers (53.75%) for the categorization of diabetes using visible tongue images. We found the classification accuracy of tongue thermal image before fusion was 90.6%, 84.3%, 83.7%, 76.8% and 72% for CNN-VGG16, SVM, LDA, *k*-NN and ResNet50 respectively. The fused tongue image provided the classification accuracy for CNN-VGG16, ResNet50, SVM, LDA, and *k*-NN as 94.3%, 78%, 88.1%, 77.8%, and 84.3%, respectively. Hence the fused image produced better classification accuracy than the individual imaging modalities. Table 6 represents the performance comparison of existing literature regarding the detection of tongue thermal imaging using machine learning and deep learning techniques.

**Table 6:** Comparative analysis of performance in existing literature regarding the detection of tongue thermal imaging using machine learning and deep learning techniques.

Author	Method	Accuracy (%)
Machine learning techniques		
Zhang et al.^27^	Digital tongue image	
	k-NN	78.77
	Naïve Bayes	75.94
	BP-NN	75
	GA-SVM	79.72
Logeswaran et al.^51^	Digital tongue image	
	SVM	66.26
Wu et al.^52^	Digital tongue image	
	k-NN	70.95
	RF	71.35
	SVM	75.20
	GA-SVM	79.72
	CNN	87.35
Deepa et al.^53^	Digital tongue image	
	DenseNet	88.29
	Dense Net-SVM	89.75
Saritha et al.^18^	Digital tongue image	
	ResNet-50	92.9
	ResNet50-RBFNN	94.4
Proposed work	Fused tongue image (digital and thermal)	
	SVM	88.12
	LDA	71.87
	k-NN	84.37
	RESNET 50	78
	VGG16	94.37

The effect of different evaporation rates on temperature measurements has been studied in both diabetic and non-diabetic subjects. The influence of evaporation on temperature measurements is not exclusive to individuals with diabetes; it is a consideration in various thermal imaging studies across diverse populations. Regarding emissivity, changes in the evaporation rate can indeed affect the apparent emissivity of the skin's surface. Emissivity is a measure of how efficiently an object emits thermal radiation. The moisture content, surface properties, and composition of the skin can be altered by evaporation, impacting its emissivity. Evaporation can change the emissivity of the skin's surface. As moisture content fluctuates due to factors such as sweating or reduced evaporation, the thermal properties of the skin may be altered, affecting the accuracy of temperature measurements. Researchers take measures to calibrate thermal cameras and control for environmental conditions to minimize the impact of factors like evaporation. However, the potential influence of evaporation is considered in the interpretation of temperature data. In summary, the effect of different evaporation rates on temperature measurements has been studied broadly, encompassing diverse populations, including non-diabetic subjects. Researchers acknowledge the potential impact of evaporation on emissivity, and they employ calibration techniques and control measures to enhance the accuracy and reliability of thermal imaging data.

The limitations of this present study are as follows: (1) the sample size is limited and might obtain a better classification accuracy rate if the sample size has been increased; (2) Generalizability of the model can be tested by incorporating various datasets obtained from various geographical locations; (3) Deploying an automated system in clinical settings can pose scalability and practicality challenges due to various factors. Primarily, clinical environments exhibit considerable diversity in size, resources, and patient demographics, complicating the adoption of a uniform solution.

In the future, we would create the three- dimensional (3D) fusion of thermal and visual tongue images to diagnose diabetes mellitus. The 3D tongue diagnostic system can be a valuable tool for obtaining a three-dimensional representation of the tongue^54^. It is particularly effective in measuring thickness and capturing rapid changes in the curvature of the tongue's surface angle. Additionally, the 3D tongue modelling scheme provides a more accurate representation. However, it's important to note that the chromatic features in tongue images contain essential disease-related information, and accurate light estimation remains a critical factor in this context.

First, the utilization of non-invasive methods for diabetes screening is crucial in enhancing early detection and intervention, which can significantly improve patient outcomes. Secondly, the use of advanced imaging techniques like fused tongue images demonstrates innovative approaches to medical diagnostics, potentially expanding the toolkit available to healthcare professionals for disease detection. However, to better contextualize the significance of these findings, it's essential for the authors to discuss the practical implications and feasibility of implementing this approach in clinical settings.

Firstly, cost-effectiveness is a key consideration, as healthcare systems often operate within constrained budgets. Understanding the cost implications of adopting this technology compared to existing screening methods is essential for decision-makers in healthcare settings. Secondly, scalability is vital for widespread adoption. The feasibility of integrating this approach into routine clinical practice across different healthcare settings needs to be explored. Factors such as the availability of equipment, training requirements for healthcare professionals, and workflow integration need to be considered. Lastly, patient acceptability is paramount for the success of any screening program. Understanding patient perceptions, concerns, and preferences regarding this novel screening method is essential for ensuring uptake and adherence. Factors such as ease of use, comfort, and potential cultural considerations should be addressed.

## Conclusions

Thus, the visible and thermal tongue thermograms are fused based on wavelet transform for the entire study population. The statistical features were extracted before and after fusing the visible and thermal tongue images with the preferred region of interest. We used the image quality metrics to evaluate the fused tongue images using nine fusion rules of DWT. Among the fusion rules, the mean-max fusion rule of DWT has outperformed the other fusion rules with lower MSE and higher PSNR values. The highest classification accuracy rate was obtained by VGG 16 (94.37%) which outperformed the other classifiers SVM (88.12%), LDA (71.87%), and *k*-NN (84.37%) for classifying the normal and diabetes using fused tongue images. Hence, this preliminary study for the fusion of thermal and visible tongue images might be used as pre-screening tools for predicting type II diabetes mellitus.

### Supplementary Information

Supplementary Information.

## Data Availability

The data that support the findings of this study are not openly available due to [reasons of sensitivity e.g. human data] and are available from the corresponding author upon reasonable request.
