# Supplementary material for: Tongue image fusion and analysis of thermal and visible images in diabetes mellitus using machine learning techniques
Source: Sci Rep. 2024 Jun 24;14:14571. doi: 10.1038/s41598-024-64150-0 (PMC11196274; doi:10.1038/s41598-024-64150-0)
Supplement: Supplementary file 1 — Supplementary Information. [file 41598_2024_64150_MOESM1_ESM.docx]

**Supplementary File**

**Tongue image Fusion and analysis of Thermal and Visible images in Diabetes Mellitus using Machine learning techniques**

Usharani Thirunavukkarasu^1,3,^ Snekhalatha Umapathy^1,2*,^ VinayaKumar Ravi^4*^, Tahani Jaser Alahmadi^5*^

^1^Department of Biomedical Engineering, College of Engineering and Technology, SRM Institute of Science and Technology, Kattankulathur, Tamil Nadu 603203 India

^2^College of Engineering, Architecture and Fine Arts, Batangas University, Batangas City, Philippines

^3^Department of Biomedical Engineering, Saveetha School of Engineering, Saveetha Institute of Medical and Technical Sciences, Chennai, Tamil Nadu 602105 India

^4^Center for Artificial Intelligence, Prince Mohammad Bin Fahd University, Khobar, Saudi Arabia

^5^Department of Information Systems, College of Computer and Information Sciences, Princess Nourah bint Abdulrahman University, P.O. Box 84428 Riyadh, Saudi Arabia

Corresponding Authors: Vinayakumar Ravi ([vinayakumarr77@gmail.com](mailto:vinayakumarr77@gmail.com) and vravi@pmu.edu.sa), Tahani Jaser Alahmadi (tjalahmadi@pnu.edu.sa), Snekhalatha Umapathy (snehalau@srmist.edu.in)

**Table S1 Wavelet Fusion Rules and Equations used in Tongue Image Fusion**

| **Fusion rule No.** | **Approximation Coefficients** | **Corresponding Details Coefficients** | **Fusion Rule Equations** |
| --- | --- | --- | --- |
| 1 | Max | Max | I_fuse_ (x,y) = W^-1^[max,max{W_T_.(IRT_1_(x,y),W_T_.(VIS_2_(x,y)}] |
| 2 | Max | Min | I_fuse_ (x,y) = W^-1^[max,min{W_T_.(IRT_1_(x,y),W_T_.(VIS_2_(x,y)}] |
| 3 | Max | Mean | I_fuse_ (x,y) = W^-1^[max,mean {W_T_.(IRT_1_(x,y),W_T_.(VIS_2_(x,y)}] |
| 4 | Mean | Max | I_fuse_ (x,y) = W^-1^[mean,max {W_T_.(IRT_1_(x,y),W_T_.(VIS_2_(x,y)}] |
| 5 | Mean | Min | I_fuse_ (x,y) = W^-1^[mean,min {W_T_.(IRT_1_(x,y),W_T_.(VIS_2_(x,y)}] |
| 6 | Mean | Mean | I_fuse_ (x,y) = W^-1^[mean,mean {W_T_.(IRT_1_(x,y),W_T_.(VIS_2_(x,y)}] |
| 7 | Min | Max | I_fuse_ (x,y) = W^-1^[min,max {W_T_.(IRT_1_(x,y),W_T_.(VIS_2_(x,y)}] |
| 8 | Min | Min | I_fuse_ (x,y) = W^-1^[min,min {W_T_.(IRT_1_(x,y),W_T_.(VIS_2_(x,y)}] |
| 9 | Min | Mean | I_fuse_ (x,y) = W^-1^[min,mean {W_T_.(IRT_1_(x,y),W_T_.(VIS_2_(x,y)}] |

I_fuse_ (x,y) – fused tongue image, IRT_1_(x,y) – Thermal tongue input image, VIS_2_ (x,y) – Visible tongue input image, W_T_ – Discrete Wavelet Transform, W^-1^ –Inverse Discrete Transform.

**Table S2 Statistical feature extraction parameters using GLCM method**

| Feature | Equation |
| --- | --- |
| Mean | $M=\frac{1}{XY}\sum_{i=1}^{X} \sum_{j=1}^{Y} G\left( i, j \right)$ |
| Standard Deviation | SD= $\sqrt{{\frac{1}{XY} \sum_{i=1}^{X} \sum_{j=1}^{Y} \left( G\left( i,j \right)-M \right)}}$ |
| Variance | Var= $\sum_{i=1}^{X} \sum_{j=1}^{Y} {(i-M)}^{2}G\left( i, j \right)$ |
| Contrast | Con= $\sum_{\text{i=0}}^{\text{H-1}} \sum_{\text{j=0}}^{\text{H-1}} \left\vert\text{i-j} \right\vert\text{2}\text{G(i,j)}.$ |
| Correlation | Corr= $\sum_{\text{i=0}}^{\text{H-1}} .\sum_{\text{j=0}}^{\text{H-1}} \frac{\text{(i.j)G(i,j)-(μ}\text{x}\text{.μ}\text{y}\text{)}}{\text{σ}\text{x}\text{.σ}\text{y}}$ |
| Energy | E= $\sqrt{\sum_{\text{i=0}}^{\text{H-1}} .\sum_{\text{j=0}}^{\text{H-1}} \text{G(i,j)}}$ |
| Entropy | Entropy= $-\sum_{\text{i=0}}^{\text{H-1}} .\sum_{\text{j=0}}^{\text{H-1}} \text{G}\left( \text{i,j} \right)\log\text{G}\text{(}\text{i,j}\text{)}$ |
| Homogeneity | $\mathrm{Hom}=\sum_{\text{j=0}}^{\text{H-1}} \frac{\text{G(i,j)}}{\text{1+\vert i-j\vert}\text{2}}$ |
| Skewness | Skewness= $\frac{[\sum{(G\left( i,j \right)-M)}^{2}/N]}{\sigma^{3}}$ |
| Kurtosis | Kurtosis= $\frac{[\sum{(G\left( i,j \right)-M)}^{4}/N]}{\sigma^{4}}-3$  Whereas,  G (i,j) – (i,j)^th^  elements of gray levels in an image.  H – Gray level quantization.  N – Number of gray levels in an image.  µ – Mean.  σ – Standard Deviation. |

**Table S3 Extracted Statistical Features using GLCM algorithm from fused tongue images by different fusion rules of DWT**

| **Fusion Rule** | **Statistical Features** | **Normal (n=80)** | **Diabetes (n=80)** | **Statistical Significance**  **(p-Value)** |
| --- | --- | --- | --- | --- |
| **Max-Max** | Contrast | 0.14±0.03 | 0.15±0.03 | p<0.01 |
|  | Energy | 0.50±0.15 | 0.49±0.13 | NS |
|  | Homogeneity | 0.929±0.01 | 0.920±0.01 | p<0.01 |
|  | Mean | 205.88±10.42 | 207.92±7.37 | NS |
|  | Standard Deviation | 10.21±1.95 | 11.05±2.47 | p<0.01 |
|  | Correlation | 0.58±0.19 | 0.57±0.18 | p<0.01 |
|  | Entropy | 5.28±0.25 | 5.37±0.30 | NS |
|  | Skewness | -2.38±2.68E | -2.38±2.68E | NS |
|  | Variance | 0.008±1.38E | 0.0008±1.40E | NS |
|  | Kurtosis | 8.13±7.15E | 8.13±7.15E | NS |
| **Max-Min** | Contrast | 0.06±0.033 | 0.05±0.04 | p<0.01 |
|  | Correlation | 0.75±0.11 | 0.72±0.22 | NS |
|  | Energy | 0.59±0.21 | 0.64±0.22 | NS |
|  | Homogeneity | 0.96±0.01 | 0.97±0.02 | p<0.01 |
|  | Mean | 206.41±10.16 | 213.04±12.18 | p<0.01 |
|  | Standard Deviation | 8.89±2.85 | 7.95±3.74 | NS |
|  | Entropy | 4.89±0.54 | 4.69±0.57 | p<0.01 |
|  | Skewness | -2.38±2.68E | -2.38±2.68E | NS |
|  | Variance | 0.008±1.38E | 0.0008±1.40E | NS |
|  | Kurtosis | 8.13±7.15E | 8.13±7.15E | NS |
| **Max-Mean** | Contrast | 0.09±0.03 | 0.15±0.04 | p<0.01 |
|  | Correlation | 0.51±0.19 | 0.55±0.13 | NS |
|  | Energy | 0.67±0.17 | 0.50±0.15 | p<0.01 |
|  | Homogeneity | 0.95±0.01 | 0.92±0.02 | p<0.01 |
|  | Mean | 196.13±13.65 | 195.37±5.39 | NS |
|  | Standard Deviation | 9.61±2.09 | 9.71±2.04 | NS |
|  | Entropy | 5.16±0.24 | 5.23±0.26 | NS |
|  | Skewness | -2.38±2.68E | -2.38±2.68E | NS |
|  | Variance | 0.008±1.38E | 0.0008±1.40E | NS |
|  | Kurtosis | 8.13±7.15E | 8.13±7.15E | NS |
| **Mean-Max** | Contrast | 0.13±0.02 | 0.07±0.04 | p<0.01 |
|  | Entropy | 5.34±0.25 | 4.84±0.51 | p<0.01 |
|  | Standard Deviation | 10.66±1.97 | 8.33±3.48 | p<0.01 |
|  | Energy | 0.48±0.12 | 0.64±0.21 | p<0.01 |
|  | Correlation | 0.52±0.16 | 0.71±0.11 | p<0.01 |
|  | Mean | 206.82±9.73 | 213.16±11.68 | p<0.01 |
|  | Variance | 0.008±1.38E | 0.0008±1.40E | NS |
|  | Homogeneity | 0.93±0.01 | 0.96±0.02 | p<0.01 |
|  | Skewness | -2.38±2.68E | -2.38±2.68E | NS |
|  | Kurtosis | 8.13±7.15E | 8.13±7.15E | NS |
| **Mean-Min** | Contrast | 0.026±0.024 | 0.05±0.02 | p<0.01 |
|  | Entropy | 4.78±0.42 | 4.88±0.39 | NS |
|  | Homogeneity | 0.98±0.01 | 0.97±0.01 | p<0.01 |
|  | Mean | 196.45±13.96 | 195.32±5.88 | NS |
|  | Standard Deviation | 7.86±3.04 | 8.21±2.38 | NS |
|  | Energy | 0.78±0.24 | 0.62±0.16 | p<0.01 |
|  | Correlation | 0.56±0.33 | 0.79±0.06 | p<0.01 |
|  | Skewness | -2.38±2.68E | -2.38±2.68E | NS |
|  | Variance | 0.008±1.38E | 0.0008±1.40E | NS |
|  | Kurtosis | 8.13±7.15E | 8.13±7.15E | NS |
| **Mean-Mean** | Contrast | 0.03±0.02 | 0.07±0.02 | p<0.01 |
|  | Correlation | 0.66±0.23 | 0.76±0.11 | p<0.01 |
|  | Skewness | -2.38±2.68E | -2.38±2.68E | NS |
|  | Energy | 0.75±0.20 | 0.58±0.16 | p<0.01 |
|  | Kurtosis | 8.13±7.15E | 8.13±7.15E | NS |
|  | Mean | 196.90±13.72 | 196.33±5.03 | NS |
|  | Standard Deviation | 8.11±2.53 | 8.41±2.03 | NS |
|  | Entropy | 4.83±0.33 | 4.98±0.33 | p<0.01 |
|  | Homogeneity | 0.97±0.01 | 0.96±0.01 | p<0.01 |
|  | Variance | 0.008±1.38E | 0.0008±1.40E | NS |
| **Min-Max** | Contrast | 0.11±0.03 | 0.17±0.01 | p<0.01 |
|  | Correlation | 0.67±0.14 | 0.70±0.11 | NS |
|  | Entropy | 5.48±0.31 | 5.72±0.30 | p<0.01 |
|  | Homogeneity | 0.94±0.06 | 0.91±0.005 | p<0.01 |
|  | Standard Deviation | 12.92±3.43 | 14.99±3.02 | p<0.01 |
|  | Mean | 184.80±19.87 | 175.26±9.64 | p<0.01 |
|  | Skewness | -2.38±2.68E | -2.38±2.68E | NS |
|  | Energy | 0.55±0.15 | 0.40±0.09 | p<0.01 |
|  | Variance | 0.008±1.38E | 0.0008±1.40E | NS |
|  | Kurtosis | 8.13±7.15E | 8.13±7.15E | NS |
| **Min-Min** | Contrast | 0.07±0.01 | 0.07±0.02 | NS |
|  | Correlation | 0.72±0.18 | 0.83±0.08 | p<0.01 |
|  | Variance | 0.008±1.38E | 0.0008±1.40E | NS |
|  | Energy | 0.60±0.18 | 0.51±0.16 | p<0.01 |
|  | Homogeneity | 0.96±0.008 | 0.96±0.014 | NS |
|  | Skewness | -2.38±2.68E | -2.38±2.68E | NS |
|  | Mean | 183.86±18.29 | 173.02±9.32 | p<0.01 |
|  | Standard Deviation | 11.36±4.39 | 13.36±3.68 | p<0.01 |
|  | Entropy | 5.21±0.32 | 5.299±0.39 | NS |
|  | Kurtosis | 8.13±7.15E | 8.13±7.15E | NS |
| **Min-Mean** | Contrast | 0.08±0.03 | 0.09±0.03 | p<0.01 |
|  | Entropy | 5.25±0.37 | 5.54±0.29 | p<0.01 |
|  | Homogeneity | 0.95±0.01 | 0.95±0.01 | p<0.01 |
|  | Standard Deviation | 11.46±4.34 | 14.30±1.35 | p<0.01 |
|  | Variance | 0.008±1.38E | 0.0008±1.40E | NS |
|  | Mean | 184.72±18.36 | 175.12±10.38 | p<0.01 |
|  | Correlation | 0.70±0.18 | 0.81±0.03 | p<0.01 |
|  | Skewness | -2.38±2.68E | -2.38±2.68E | NS |
|  | Energy | 0.059±0.17 | 0.47±0.11 | p<0.01 |
|  | Kurtosis | 8.13±7.15E | 8.13±7.15E | NS |

p<0.01 – significant, NS- Not Significant

**Table S4 Image Quality Metrics Assessment after the tongue image fusion using nine different combinations of fusion rules using DWT**

| **Fusion rules** | **Fused Tongue (n=160)** | **MSE** | **PSNR** | **SNR** | **NAE** | **NCC** | **AD** | **MD** | **SC** | **SSIM** |
| --- | --- | --- | --- | --- | --- | --- | --- | --- | --- | --- |
| **Max-Max** | Normal | 585.76 | 20.67 | 20.22 | 0.03 | 1.01 | -8.58 | 74.82 | 0.95 | 0.96 |
|  | Diabetes | 551.78 | 20.99 | 20.57 | 0.039 | 1.02 | -8.02 | 75.22 | 0.95 | 0.96 |
| **Max-Min** | Normal | 962.31 | 19.14 | 18.71 | 0.042 | 1.02 | -10.84 | 87.14 | 0.94 | 0.95 |
|  | Diabetes | 787.67 | 19.65 | 19.24 | 0.043 | 1.02 | -9.55 | 81.33 | 0.94 | 0.95 |
| **Max-Mean** | Normal | 793.17 | 19.83 | 19.38 | 0.045 | 1.02 | -9.78 | 53.8 | 0.94 | 0.94 |
|  | Diabetes | 725.67 | 20.02 | 19.60 | 0.041 | 1.02 | -9.27 | 45.747 | 0.94 | 0.94 |
| **Mean-Max** | Normal | 180.47 | 25.65 | 25.01 | 0.021 | 1.00 | -1.05 | 100.61 | 0.99 | 0.98 |
|  | Diabetes | 233.36 | 24.57 | 23.95 | 0.026 | 0.09 | -0.50 | 97.08 | 0.99 | 0.98 |
| **Mean-Min** | Normal | 230.18 | 24.62 | 23.97 | 0.024 | 1.00 | -1.38 | 110.83 | 0.99 | 0.95 |
|  | Diabetes | 292.32 | 23.55 | 22.92 | 0.029 | 0.99 | -0.93 | 107.84 | 0.99 | 0.95 |
| **Mean-Mean** | Normal | 187.66 | 25.51 | 24.85 | 0.022 | 1.00 | -1.55 | 76.48 | 0.99 | 0.96 |
|  | Diabetes | 234.39 | 24.57 | 23.96 | 0.026 | 0.99 | -0.74 | 76.64 | 0.99 | 0.96 |
| **Min-Max** | Normal | 385.81 | 22.56 | 21.75 | 0.031 | 0.97 | 6.83 | 142.48 | 1.03 | 0.93 |
|  | Diabetes | 534.39 | 21.45 | 20.66 | 0.034 | 0.97 | 7.55 | 129.64 | 1.03 | 0.93 |
| **Min-Min** | Normal | 413.65 | 22.16 | 21.35 | 0.032 | 0.98 | 6.60 | 153.94 | 1.03 | 0.96 |
|  | Diabetes | 543.72 | 21.25 | 20.45 | 0.036 | 0.97 | 7.26 | 157.64 | 1.03 | 0.96 |
| **Min-Mean** | Normal | 381.95 | 22.59 | 21.78 | 0.031 | 0.97 | 6.72 | 128.25 | 1.06 | 0.95 |
|  | Diabetes | 518.88 | 21.47 | 20.66 | 0.035 | 0.97 | 7.43 | 132.74 | 1.03 | 0.95 |


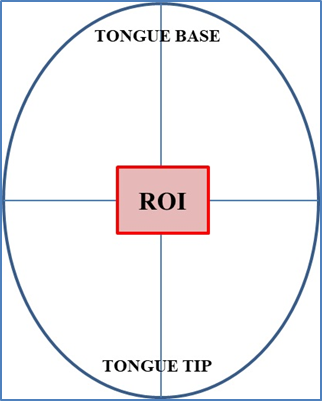


**Figure S1 ROI indicating Middle part of the Tongue**
